# Molecular identification of a new species of *Rhigonema* (Nematoda: Rhigonematidae) and phylogenetic relationships within the infraorder Rhigonematomorpha

**DOI:** 10.1186/s13071-022-05544-9

**Published:** 2022-11-15

**Authors:** Yu Zhang, Lian-Di Wang, Koichi Hasegawa, Seiya Nagae, Hui-Xia Chen, Lin-Wei Li, Liang Li

**Affiliations:** 1 Hebei Key Laboratory of Animal Physiology, Biochemistry and Molecular Biology, College of Life Sciences, Hebei Normal University, Shijiazhuang, Hebei Province People’s Republic of China; 2 Hebei Collaborative Innovation Center for Eco-Environment, Shijiazhuang, Hebei Province People’s Republic of China; 3 Key Laboratory of Molecular Cell Biology, Ministry of Education of the People’s Republic of China , Hebei Normal University, Shijiazhuang, 050024 Hebei Province People’s Republic of China; 4 Department of Environmental Biology, College of Bioscience & Biotechnology, Chubu University, Aichi, Japan

**Keywords:** Parasite, Nematoda, Rhigonematomorpha, DNA taxonomy, Genetic data, Species delimitation, Molecular phylogeny, Millipede

## Abstract

**Background:**

The infraorder Rhigonematomorpha comprises a group of obligate parasitic nematodes of millipedes (Arthropoda: Diplopoda). The current species identification of Rhigonematomorpha nematodes remains mainly based on morphological features, with molecular-based identification still in its infancy. Also, current knowledge of the phylogeny of Rhigonematomorpha is far from comprehensive.

**Methods:**

The morphology of Rhigonematomorpha nematodes belonging to the genus *Rhigonema*, collected from the millipede *Spirobolus bungii* Brandt (Diplopoda: Spirobolida) in China, was studied in detail using light and scanning electron microscopy. Five different genetic markers, including the nuclear small ribosomal subunit (18S), internal transcribed spacer (ITS) and large ribosomal subunit (28S) regions and the mitochondrial *cox*1 and *cox*2 genes of these Rhigonematomorpha nematodes collected from China and *Rhigonema naylae* collected from Japan were sequenced and analyzed using Bayesian inference (BI) and Assemble Species by Automatic Partitioning (ASAP) methods. Phylogenetic analyses that included the most comprehensive taxa sampling of Rhigonematomorpha to date were also performed based on the 18S + 28S genes using maximum likelihood (ML) and BI methods.

**Results:**

The specimens of *Rhigonema* collected from *S. bungii* in China were identified as a new species, *Rhigonema sinense* n. sp. Striking variability in tail morphology was observed among individuals of *R. sinense* n. sp. ASAP analyses based on the 28S, ITS, *cox*1 and *cox*2 sequences supported the species partition of *R. sinense* n. sp. and *R. naylae*, but showed no evidence that the different morphotypes of *R. sinense* n. sp. represent distinct genetic lineages. BI analyses also indicated that *R. sinense* n. sp. represents a separated species from *R. naylae* based on the *cox*1 and *cox*2 genes, but showed that *R. naylae* nested in samples of *R. sinense* n. sp. based on the ITS and 28S data. Phylogenetic results showed that the representatives of Rhigonematomorpha formed two large clades. The monophyly of the families Carnoyidae and Ichthyocephalidae and the genus *Rhigonema* was rejected. The representatives of the family Ransomnematidae clustered together with the family Hethidae with strong support.

**Conclusions:**

A new species of Rhigonematomorpha, *R. sinense* n. sp. is described based on morphological and molecular evidence. ASAP analyses using 28S, ITS, *cox*1 and *cox*2 data indicate the striking variability in tail morphology of *R. sinense* n. sp. as intraspecific variation, and also suggest that partial 28S, ITS, *cox*1 and *cox*2 markers are effective for molecular identification of Rhigonematomorpha nematodes. The phylogenetic results support the traditional classification of Rhigonematomorpha into the two superfamilies Rhigonematoidea and Ransomnematoidea, and indicate that the families Carnoyidae and Ichthyocephalidae and the genus *Rhigonema* are non-monophyletic. The present phylogeny strongly supports resurrection of the family Brumptaemiliidae, and also indicates that the family Ransomnematidae is sister to the family Hethidae.

**Graphical Abstract:**

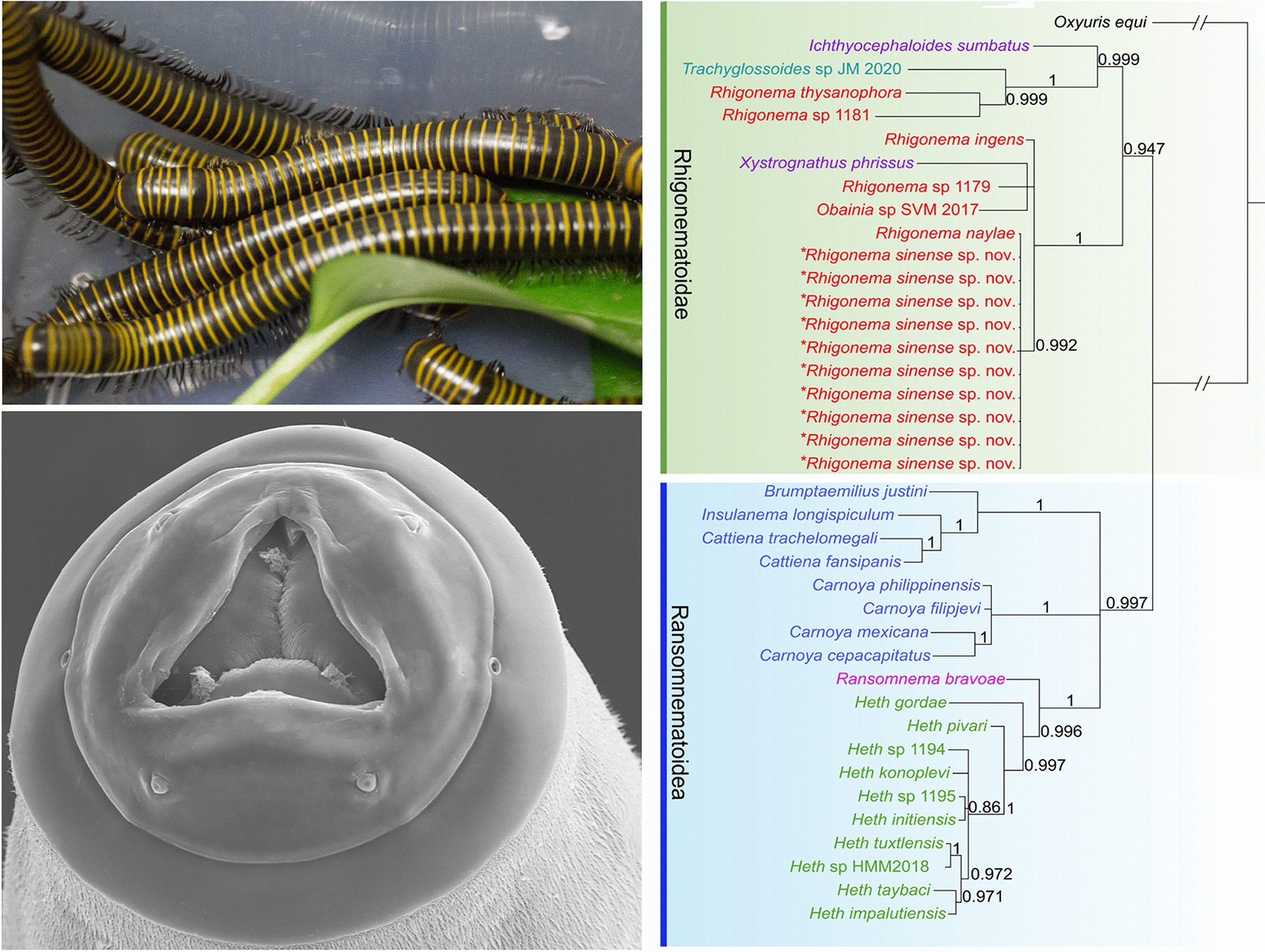

## Background

Nematodes of the infraorder Rhigonematomorpha are obligate endoparasites of millipedes (Arthropoda: Diplopoda) with monoxenous life-cycles [1]. To date, over 200 nominal species of Rhigonematomorpha have been described worldwide [2, 3]. According to the current classifications, which are mainly based on morphological characters, Rhigonematomorpha is divided into six families assigned into two superfamilies, namely Rhigonematoidea (Rhigonematidae, Ichthyocephalidae, Xustromatidae) and Ransomnematoidea (Carnoyidae, Hethidae, Ransomnematidae) [1, 2, 4]. However, the monophyly and phylogenetic relationships of these six families are still under debate [3, 5–7].

The current species identification of Rhigonematomorpha nematodes remains mainly based on morphological features [8–13]. However, it is not easy to distinguish some congeners only using morphology due to their high similarities. Furthermore, the morphology-based method is not able to effectively delimit the phenotypic plasticity and discover sibling or cryptic species.

Several recent studies have provided some nuclear and mitochondrial (mt) DNA sequence data [i.e. the small subunit ribosomal DNA (18S), the large subunit ribosomal DNA (28S) and the mitochondrial cytochrome* c* oxidase subunit 1 (*cox*1) gene or the mitochondrial genome] that can be used for species identification or phylogeny of Rhigonematomorpha [6, 14–19]. However, the current genetic database for these nematodes remains very limited. In Rhigonematomorpha, only 21 nominal species have been genetically characterized [14–17, 19, 20], and most of the data available are represented by the 18S and 28S sequences, which are commonly used for molecular phylogeny of higher taxa within Nematoda [21–26]. Although the nuclear internal transcribed spacer (ITS) region and the mitochondrial *cox*1 and *cox*2 genes are widely used as powerful and practical genetic markers for revealing sibling or cryptic species, delimiting phenotypic variation and identifying species in the infraorders Ascaridomorpha, Spiruromorpha and Oxyuridomorpha [27–44], they have been scarcely employed in studies pertaining to Rhigonematomorpha species. Consequently, no current knowledge on the effectiveness of ITS, *cox*1 and *cox*2 as genetic markers for identification of Rhigonematomorpha nematodes is available.

In the present study, a large number of Rhigonematomorpha nematodes belonging to the genus *Rhigonema* (Rhigonematoidea: Rhigonematidae) were collected from the millipede *Spirobolus bungii* Brandt (Diplopoda: Spirobolida) in China. Striking variability in the morphology of tail in both male and female specimens was observed among different individuals in the study material. In order to compare the suitability and efficacy of different nuclear and mitochondrial genetic markers for delimitation of the phenotypic variation of different individuals and discrimination of the morphologically similar Rhigonematomorpha congeners, the nuclear 18S, ITS and 28S regions and the mitochondrial *cox*1 and *cox*2 genes of the present specimens collected from China and *R. naylae* Morffe & Hasegawa, 2017 collected from *Parafontaria tonominea* (Polydesmida: Xystodesmidae) in Japan were sequenced and analyzed using Bayesian inference (BI) and Assemble Species by Automatic Partitioning (ASAP) methods. Furthermore, in order to test the monophyly and evaluate the evolutionary relationships of the six families within Rhigonematomorpha, we performed phylogenetic analyses, including the most comprehensive taxa sampling of Rhigonematomorpha to date, based on the 18S + 28S genes using maximum likelihood (ML) and BI.

## Methods

### Light and scanning electron microscopy

Nematodes were collected from the hindgut of the millipede *S. bungii* in Shijiazhuang, Hebei Province, China, and the specimens fixed and stored in 80% ethanol until study. For the light microscopy studies, nematodes were cleared in glycerin for examination using a Nikon® optical microscope (Nikon Corp., Tokyo, Japan). Photomicrographs were recorded using a Nikon® digital camera coupled to a Nikon® optical microscope (Nikon ECLIPSE Ni-U; Nikon Corp.). For scanning electron microscopy (SEM), specimens were re-fixed in a 4% formaldehyde solution, post-fixed in 1% OsO_4_, dehydrated via an ethanol series (50%, 70%, 80%, 90%, 100%, 100%) and acetone (100%) and then critical point dried. Samples were coated with gold and examined using a Hitachi S–4800 scanning electron microscope (Hitachi Ltd., Tokyo, Japan) at an accelerating voltage of 20 kV. Measurements (range with mean in parentheses) are given in micrometers unless otherwise stated. Type specimens were deposited in College of Life Sciences, Hebei Normal University, Hebei Province, China.

### Molecular procedures

The mid-body of 10 selected nematode specimens (4 males, 6 females) with a different morphology of the tail tip, all specimens collected from *S. bungii* in China, was used for molecular analysis (Table 1). Genomic DNA from each sample was extracted using a Column Genomic DNA Isolation Kit [Sangon Biotech (Shanghai) Co., Ltd., Shanghai, China] according to the manufacturer’s instructions. The partial 18S region was amplified by PCR using the forward primer Nem_18S_F (5′-CGC GAA TRG CTC ATT ACA ACA GC-3′) and the reverse primer Nem_18S_R (5′-GGG CGG TAT CTG ATC GCC-3′) [45]. The partial 28S region was amplified by PCR using the forward primer D2a (5′-ACA AGT ACC GTG AGG GAA AGT TG-3′) and the reverse primer D3b (5′-TCG GAA GGA ACC AGC TAC TA-3′) [46]. The ITS-1 region was amplified by PCR using the forward primer (5′-AGC GGG GAC TGC TGT TTC GAT ACC TTT CGG-3′) and the reverse primer (5′-GTT CGA CCC TCA GCC AGA CGT GCC AAG GGG-3′) designed in the present study. The ITS-2 region was amplified by PCR using the forward primer (5′-CTA CTC TTA GCG GTG GAT CAC TCG GCT CGT-3′) and the reverse primer (5′-TCT AGC ACC TTC TAT GGA CTG TAG CCC CGC-3′) designed in the present study. The partial *cox*1 region was amplified by PCR using the forward primer LCO (5′-GGT CAA CAA ATC ATA AAG ATA TTG G -3′) and the reverse primer HCO (5′-TAA ACT TCA GGG TGA CCA AAA AAT CA -3′) [47]. The partial *cox*2 region was amplified by PCR using the forward primer (5′-ATG AAA TTT CCA ATT TTG AGG CTT ATA GGG-3′) and the reverse primer (5′-ATA AAC TAA AAA GCT AAA AAT TAT TAA AAA-3′) designed in the present study.

**Table 1:** Specimens of *Rhigonema sinense* n. sp. selected for molecular analysis

Samples (specimen no.)	Genbank accession numbers of partial 18S region	Genbank accession numbers of partial 28S region	Genbank accession numbers of ITS region	Genbank accession numbers of partial* cox*1 region	Genbank accessions numbers of partial* cox*2 region	Morphotypes
1 female (49)	ON936095	ON936078	ON936109	ON935732	OP157155	Without finger-like tail tip (Fig. 3g)
1 female (43)	ON938178	ON936079	ON936110	ON935729	OP157154	Without finger-like tail tip (Fig. 3g)
1 female (69)	ON938172	ON936082	ON936112	OP159049	OP157157	Long finger-like tail tip (Fig. 3e)
1 female (20)	ON938182	ON936083	ON936106	ON935601	OP157153	Long finger-like tail tip (Fig. 3e)
1 female (68)	ON936087	ON936077	ON936104	OP103756	OP157162	Short finger-like tail tip (Fig. 3f)
1 female (51)	ON938174	ON936080	ON936111	ON935744	OP157156	Short finger-like tail tip (Fig. 3f)
1 male (71)	ON936088	ON936081	ON936108	ON935613	OP157158	Long finger-like tail tip (Fig. 3c)
1 male (37)	ON938171	ON936086	ON936107	OP103757	OP157161	Long finger-like tail tip (Fig. 3c)
1 male (67–1)	ON937754	ON936084	ON936105	ON935751	OP157159	Short finger-like tail tip (Fig. 3h)
1 male (67–2)	ON938173	ON936085	–	OP103758	OP157160	Short finger-like tail tip (Fig. 3h)

*cox1/2* Cytochrome* c* oxidase subunit 1/2,* ITS* internal transcribed spacer, *18S/28S* small/large ribosomal subunit

Samples of *R. naylae* collected from the millipede *P. tonominea* in Japan were also used for molecular analysis. The partial ITS and *cox*1 regions of *R. naylae* were amplified by PCR using the same primers as mentioned above for the specimens collected from *S. bungii* in China. The partial *cox*2 region of *R. naylae* was amplified by PCR using the forward primer Rhigo_COXII_For (5′-TCH ACY ACA ATA GGY ATA AAM CT-3′) and the reverse primers Rhigo_COII_Rev (5′-GWT ATA TRG RTT GGT TYC ATA A-3′), as well as by Rhigo_COII_RevNtd (5′-GGT TYC ATA ATT TTA MTT RTA G-3′) designed in the present study.

All PCR assays of nematodes collected from *S. bungii* in China were performed in a 50-μl volume containing PCR reaction buffer with 10 mM Tris HCl at pH 8.4, 50 mM KCl, 3.0 mM MgCl_2_, 250 μM of each dNTP, 50 pmol of each primer and 1.5 U of Taq polymerase (Takara Bio Inc., Kusatsu, Shiga, Japan) in a thermocycler (model 2720; Applied Biosystems, Thermo Fisher Scientific, Waltham, MA, USA). The cycling conditions for the different regions were:

 Partial 18S region: an initial denaturation at 94 °C for 5 min, followed by 35 cycles at 94 °C, 30 s (denaturation), 52 °C, 40 s (annealing) and 72 °C, 60 s (extension), with a final extension of 72 °C for 10 min

Partial 28S region: an initial denaturation at 94 °C for 5 min, followed by 35 cycles of 94 °C, 30 s (denaturation), 56 °C, 30 s (annealing) and 72 °C, 70 s (extension), with a final extension of 72 °C for 7 min

 Partial ITS region: an initial denaturation at 94 °C for 5 min, followed by 30 cycles of 94 °C, 30 s (denaturation), 68 °C, 30 s (annealing) and 72 °C, 20 s (extension), with a final extension of 72 °C for 7 min

 Partial *cox*1 region: an initial denaturation at 95 °C for 5 min, followed by 35 cycles of 95 °C, 30 s (denaturation), 50 °C, 30 s (annealing) and 72 °C, 60 s (extension), with a final extension of 72 °C for 10 min

 Partial *cox*2 region: an initial denaturation at 94 °C for 5 min, followed by 35 cycles of 94 °C, 30 s (denaturation), 46 °C, 30 s (annealing) and 72 °C, 60 s (extension), with a final extension of 72 °C for 10 min.

All PCRs of samples of *R. naylae* collected from *P. tonominea* in Japan were performed in a 20-μl volume of PCR reaction buffer containing 20 mM Tris HCl at pH 7.5, 8.0 mM MgCl_2_, 400 μM of each dNTP, 0.3 μM of each primer and 0.02 U/μl of KOD FX Neo DNA polymerase (Toyobo Co. Ltd., Osaka, Osaka, Japan) in a thermocycler (model Dice® Touch; Takara Bio Inc.). The cycling conditions for the different regions were:Partial ITS and *cox*1 regions: an initial denaturation at 94 °C for 1 min, followed by 35 cycles of 98 °C, 10 s (denaturation), 55 °C, 30 s (annealing) and 68 °C, 1 min (extension), with a final extension of 68 °C for 5 min

Partial *cox*2 regions: A first PCR was performed with primes Rhigo_COXII_For and Rhigo_COXII_Rev under the following the conditions: an initial denaturation at 94 °C for 1 min, followed by 35 cycles of 98 °C, 10 s (denaturation), 40 °C, 30 s (annealing) and 68 °C, 1 min (extension), with a final extension of 68 °C for 5 min. Then, nested PCR was performed with primers Rhigo_COXII_For and Rhigo_COXII_RevNtd, using 3 μl of the first PCR product as template. PCR conditions were same as those of the first PCR reaction.

PCR products were checked on GoldView-stained 1.5% agarose gels and purified with the Column PCR Product Purification Kit [Sangon Biotech (Shanghai) Co., Ltd.]. Sequencing for each sample was carried out on both strands. Sequences were aligned using ClustalW2. The DNA sequences obtained herein were compared (using the algorithm BLASTn) with those available in the National Center for Biotechnology Information (NCBI) database (http://www.ncbi.nlm.nih.gov). The 18S, 28S, ITS, *cox*1 and *cox*2 sequence data of specimens collected from China and *R. naylae* collected from Japan were deposited in the GenBank (http://www.ncbi.nlm.nih.gov).

### Species delimitation

The BI and ASAP [48] methods were employed for species delimitation of *Rhigonema* spp. based on the 18S, 28S, ITS, *cox*1 and *cox*2 sequences, respectively. The BI trees were inferred using MrBayes 3.2.7 [49] under the JC model for each genetic marker (two parallel runs, 1,000,000 generations). *Rhigonema thysanophora* (Rhigonematomorpha: Rhigonematoidea) and *Krefftascaris sharpiloi* (Ascaridida: Ascaridoidea) were chosen as outgroups. The ASAP analyses were conducted using the ASAP online server (https://bioinfo.mnhn.fr/abi/public/asap) under the Kimura (K80) ts/tv model. The results of ASAP with the lowest scores were considered to be the optimal group number, with the exception of the optimal results recommended by ASAP.

### Phylogenetic analyses

Phylogenetic analyses were performed based on the 18S + 28S sequence data using ML inference with IQTREE v2.1.2 [50] and BI with MrBayes 3.2.7 [49], respectively. *Oxyuris equi* (Oxyurida: Oxyuroidea) was chosen as the out-group. The in-group included 28 representatives of Rhigonematomorpha representing all six families belonging to the two superfamilies Rhigonematoidea and Ransomnematoidea. Detailed information on the Rhigonematomorpha nematodes included in the present phylogenetic analyses is provided in Table 2.

**Table 2:** Detailed information on Rhigonematomorpha nematodes with their genetic data included in the phylogenetic analyses

Species	Host	Locality	Accession numbers for 18S region	Accession numbers for 28S region	References
*Ingroup Rhigonematoidea*					
* Rhigonema thysanophora*	*Euryurus* sp.	USA	EF180067.1	MG195996.1	[70]
* Rhigonema naylae*	*Parafontaria laminate*	Japan	KX844642.1	KX844643.1	[17]
* Rhigonema ingens*	*Thyropygus* sp.	Vietnam	JX069475.1	JX131616.1	[7]
* Rhigonema* sp. 1179	*Apeuthes* sp.	Vietnam	JX106453.1	JX155275.1	[7]
* Rhigonema* sp. 1181	*Apeuthes* sp.	Vietnam	JX106455.1	JX155276.1	[7]
* Rhigonema sinense*	*Spirobolus bungii*	China	ON936095	ON936078	Present study
* Rhigonema sinense*	*Spirobolus bungii*	China	ON938178	ON936079	Present study
* Rhigonema sinense*	*Spirobolus bungii*	China	ON938172	ON936082	Present study
* Rhigonema sinense*	*Spirobolus bungii*	China	ON938182	ON936083	Present study
* Rhigonema sinense*	*Spirobolus bungii*	China	ON936087	ON936077	Present study
* Rhigonema sinense*	*Spirobolus bungii*	China	ON938174	ON936080	Present study
* Rhigonema sinense*	*Spirobolus bungii*	China	ON936088	ON936081	Present study
* Rhigonema sinense*	*Spirobolus bungii*	China	ON938171	ON936086	Present study
* Rhigonema sinense*	*Spirobolus bungii*	China	ON937754	ON936084	Present study
* Rhigonema sinense*	*Spirobolus bungii*	China	ON938173	ON936085	Present study
* Obainia* sp. SVM-2017	*Archispirostreptus gigas*	Tanzania	KU561101.1	KU561100.1	[64]
* Ichthyocephaloides sumbatus*	*Salpidobolus* sp.	Indonesia	JX101958.1	JX155273.1	[7]
* Xystrognathus phrissus*	*Apeuthes* sp.	Vietnam	JX101957.1	JX155274.1	[7]
* Trachyglossoides* sp.	*Spirobolellus* sp.	Cuba	MW030192.1	MW030188.1	Unpublished
*Ransomnematoidea*					
* Ransomnema bravoae*	*Anadenobolus putealis*	Mexico	KY857887.1	KY857886.1	[3]
* Carnoya mexicana*	*Anadenobolus putealisLoomis*	Mexico	KT236089.1	KT236088.1	[63]
* Carnoya cepacapitatus*	*Anadenobolus putealisLoomis*	Mexico	KT236087.1	KT236086.1	[63]
* Carnoya filipjevi*	*Salpidobolus* sp.	Indonesia	JX982120.1	JX946703.1	[62]
* Carnoya philippinensis*	Rhinocricidae sp.	Philippines	KT957946.1	KT957945.1	[71]
* Cattiena trachelomegali*	*Thyropygus* sp.	Vietnam	JX982117.1	JX419378.1	[5]
* Cattiena fansipanis*	Pseudospirobolellidae sp.	Vietnam	JX982118.1	JX436470.1	[5]
* Brumptaemilius justini*	*Archispirostreptus gigas*	Tanzania	JX999733.1	JX999732.1	[5]
* Insulanema longispiculum*	*Apeuthes* sp.	Vietnam	JX982119.1	JX436471.1	[5]
* Heth taybaci*	Harpagophoridae sp.	Vietnam	JX987085.1	JX946704.1	[5]
* Heth impalutiensis*	Spirosreptidae sp.	Philippines	KM226161.1	KM226162.1	[6]
* Heth tuxtlensis*	*Anadenobolus putealis*	Mexico	KY857883.1	KY857884.1	[3]
* Heth konoplevi*	Rhinocricidae sp.	Philippines	KY985469.1	KY985470.1	[64]
* Heth initiensis*	Rhinocricidae sp.	Philippines	KY985471.1	KY985472.1	[64]
* Heth pivari*	*Narceus gordanus*	USA	MK182092.1	MK182091.1	[18]
* Heth gordae*	*Anadenobolus putealis*	Mexico	KY857879.1	KY857880.1	[3]
* Heth* sp. 1 HMM2018	*Anadenobolus putealis*	Mexico	KY857881.1	KY857882.1	[3]
* Heth* sp. 1195	*Salpidobolus* sp.	Indonesia	JX987087.1	JX443483.1	[5]
* Heth* sp. 1194	*Spirostreptida* sp.	Australia	JX987086.1	JX443484.1	[5]
*Outgroup*					
* Oxyuris equi*	–	–	KU180664.1	KU180675.1	[72]

The nucleotide sequences were aligned in batches using MAFFT v7.313 with the iterative refinement method of E-INS-I [51]; poorly aligned regions were excluded using BMGE v1.12 (*h* = 0.4) [52]. In addition, partially ambiguous bases were manually inspected and removed. Substitution models were compared and selected according to the Bayesian information criterion (BIC) by using ModelFinder [53]. The TIM3e + I + G4 model in ML inference and the SYM + I + G model in BI were identified as the optimal nucleotide substitution model, respectively. Reliabilities for ML inference were tested using 1000 bootstrap replications, and BIC analysis was run for 5 × 106 Markov chain Monte Carlo (MCMC) generations.

In the ML tree, the bootstrap (BS) values ≥ 90 were considered to constitute strong nodal support, whereas BS values ≥ 70 and < 90 were considered to constitute moderate nodal support. In the BI tree, the Bayesian posterior probabilities (BPP) values ≥ 0.90 were considered to constitute strong nodal support, whereas BPP values ≥ 0.70 and < 0.90 were considered to constitute moderate nodal support. BS values ≥ 70 and BPP values ≥ 0.70 are shown in the phylogenetic trees.

## Results

**Order Spirurida Railliet 1914**

**Infraorder Rhigonematomorpha De Ley & Blaxter, 2002**

**Family Rhigonematidae Artigas 1930**

**Genus** ***Rhigonema*** **Cobb, 1898**

***Rhigonema sinense*** **Zhang, Wang, Hasegawa, Nagae, Chen, Li & Li n. sp.**

***Type-host*:** *Spirobolus bungii* (Brandt) (Spirobolida: Spirobolidae).

***Type-locality***: Shjiazhuang, Hebei Province, China.

***Site in host***: Hindgut.

***Type specimens***: Holotype, male (HBNU-N-2022Ar008Z-L); allotype, female (HBNU-N-2022Ar009Z-L); paratypes: 16 males, 16 females (HBNU-N-2022Ar010Z-L); deposited in the College of Life Sciences, Hebei Normal University, Hebei Province, China.

***Representative DNA sequences***: Representative nuclear ribosomal and mitochondrial DNA sequences were deposited in the GenBank database under the accession numbers ON936087, ON936088, ON936095, ON937754, ON938171–ON938174, ON938178, ON938182 (18S), ON936077–ON936086 (28S), ON936104–ON936112 (ITS), OP159049, OP103756–OP103758, ON935601, ON935613, ON935729, ON935732, ON935744, ON935751 (*cox*1) and OP157153–OP157162 (*cox*2).

***ZooBank registration***: To comply with the regulations set out in article 8.5 of the amended 2012 version of the International Code of Zoological Nomenclature (ICZN), details of the new species have been submitted to ZooBank. The Life Science Identifier (LSID) of the article is urn:lsid:zoobank.org:pub: 16047F5E-A719-4A63-9F8F-260AA4345341. The LSID for the new name *Rhigonema sinense* is urn:lsid:zoobank.org:act: D7722213-B0FD-450F-B891-619A97ECC90B*.*

***Etymology***: The specific name refers to its geographic origin (China), which represents the first new species of Rhigonematomorpha described in China.

### Description

#### General

**Fig. 1:**
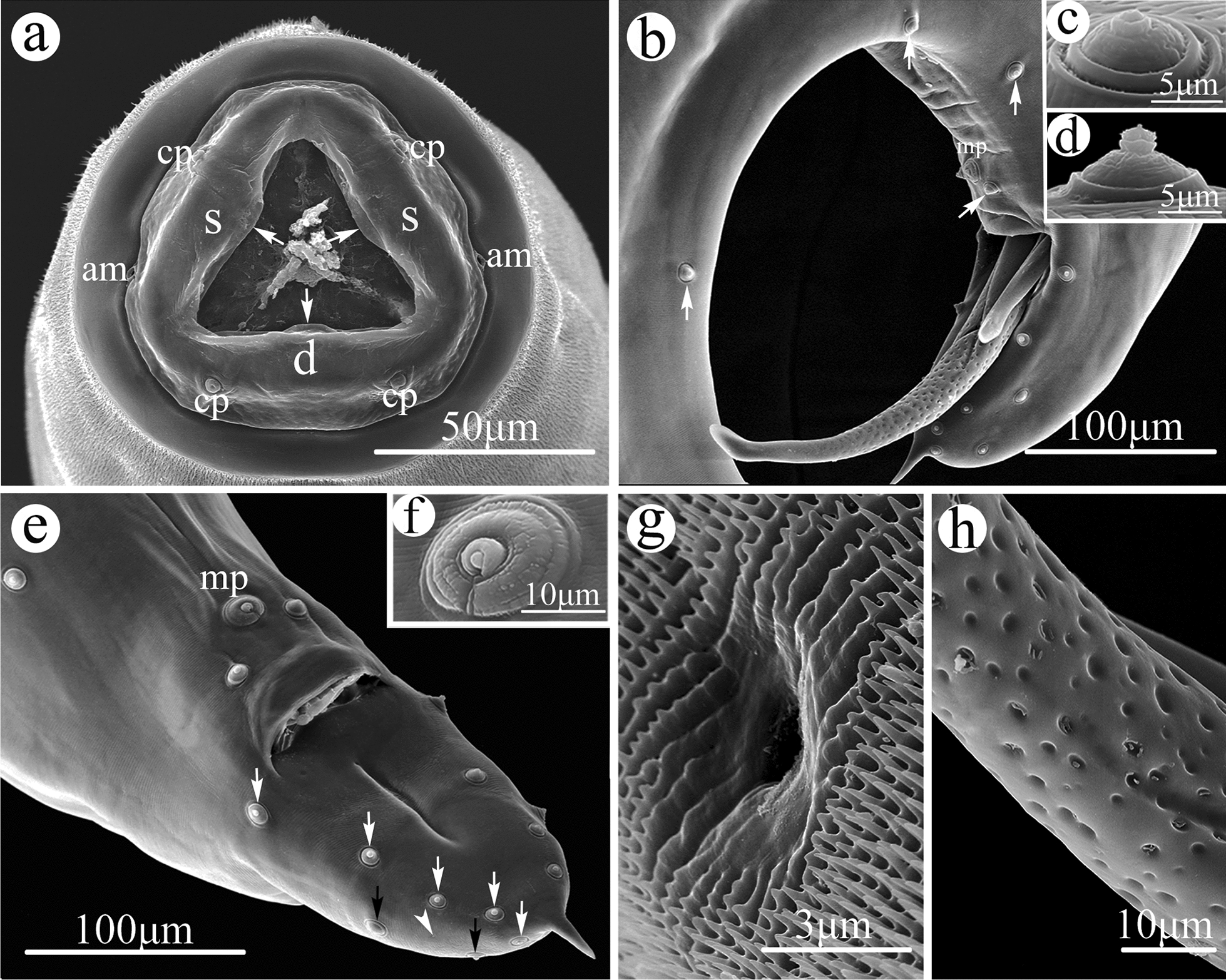
Scanning electron micrographs of the nematode *Rhigonema sinense* n. sp. collected from the millipede *Spirobolus bungii* (Spirobolida: Spirobolidae) in China, male.** a** Cephalic end (arrows: inner lip margins), apical view.** b** Posterior end of body, lateral view (arrows: precloacal papillae ).** c**–**h** Magnified images of precloacal papilla (**c**), postcloacal papilla (**d**), tail (arrows: postcloacal papillae and phasmid) (**e**), single median precloacal papilla (**f**), excretory pore and small spines (microtrichs) (**g**) and spicule, showing surface sculptured with randomly scattered punctations (**h**). am, Amphids; cp, cephalic papillae; d, dorsal lip; mp, median precloacal papilla; s, sub-ventral lip

**Fig. 2:**
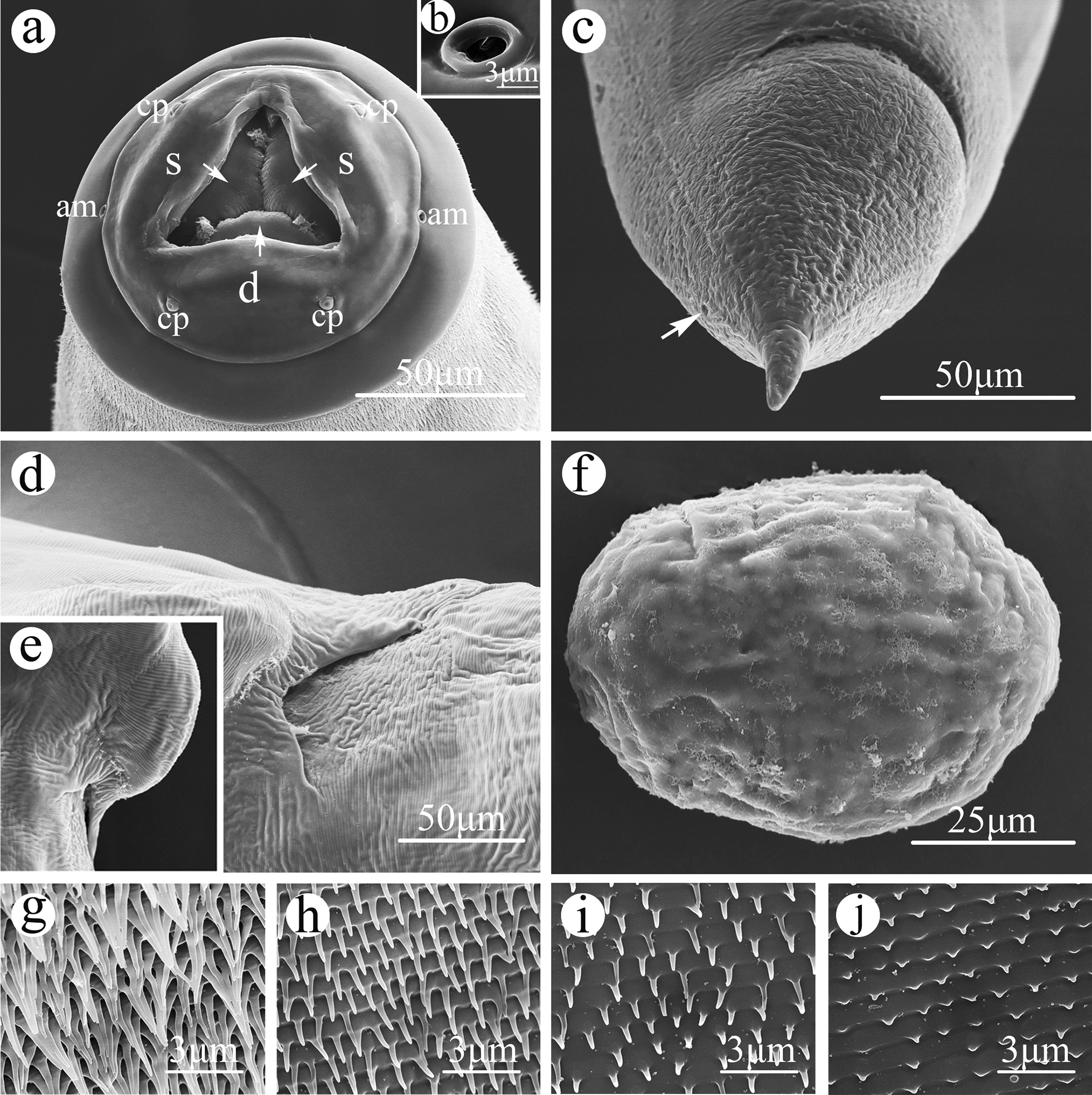
Scanning electron micrographs of *Rhigonema sinense* n. sp. collected from *Spirobolus bungii* (Spirobolida: Spirobolidae) in China, female.** a** Cephalic end (arrows: flabellate pharyngeal plates), apical view.** b–i** Magnified images of amphidial aperture (**b**), posterior end of female (arrow: phasmid) (**c**), vulva (**d**,** e**), egg (**f**), cuticular spines (microtrichs) (**g**–**j**). For abbreviations, see Fig. 1 caption

**Fig. 3:**
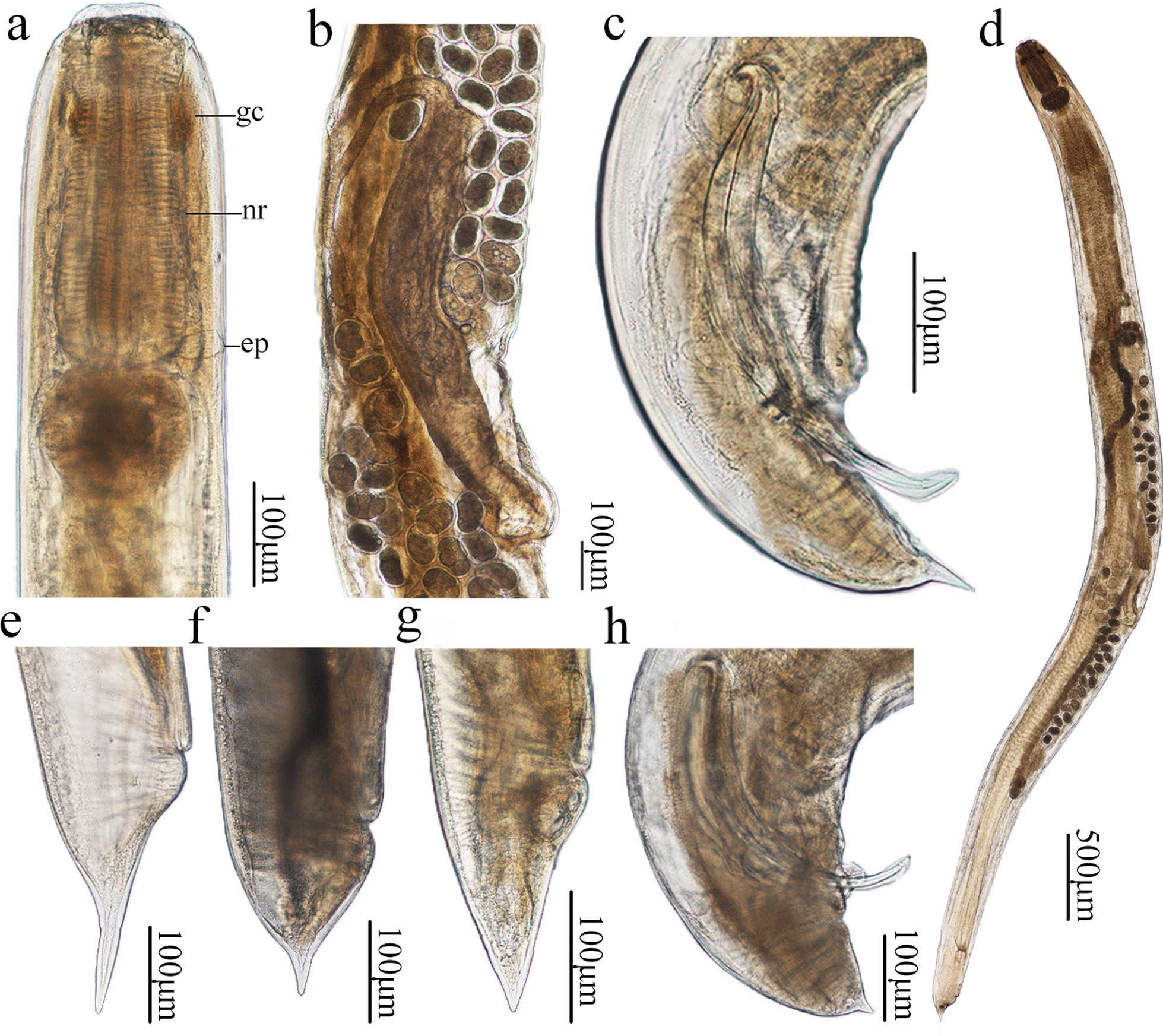
Photomicrographs of *Rhigonema sinense* n. sp. collected from *Spirobolus bungii* (Spirobolida: Spirobolidae) in China.** a** Anterior part of male, lateral view,** b** region of vulva, lateral view,** c**, **d** body of female, lateral view,** e**–**g** tail of female, lateral view, **h** posterior end of male, lateral view. ep, Excretory pore; gc, glandular cell; nr, nerve ring

 Small-sized, whitish nematodes with a maximum width at about mid-body. Cephalic region heavily cuticularized, consisting of well-developed cephalic cap and smooth cephalic collar (Figs. 1a, 2a). Cephalic cap bearing three apparent lips, dorsal lip with one pair of large cephalic papillae, subventral lips with a single large cephalic papilla each, amphidial apertures located laterally at junction of cephalic cap and cephalic collar (Figs. 1a, 2a); each lip with unconspicuous inner lip margins (Fig. 1a). Oral aperture simple, somewhat triangular (Figs. 1a, 2a). Cuticle posterior to cephalic region with dense, transverse rows of small spines (microtrichs); rows of spines gradually becoming distinctly sparser and smaller towards posterior region of body and disappearing at about the anterior 1/4 region of body (Figs. 1a, g, 2a, g–j). Esophagus divided into short chitinized pharynx with three flabellate pharyngeal plates (Fig. 2a), muscular cylindrical corpus (posterior part slightly wider than anterior part), unconspicuous isthmus and ovoid or nearly rounded posterior bulb (Fig. 3a, d). Nerve ring at about 1/2 of esophageal corpus (Fig. 3a). Excretory pore at about junction of corpus and posterior bulb of esophagus (Figs. 1g, 3a). Tail of both sexes conical, with polymorphic tip (Figs. 1b, e, 2c, 3c–h).

#### Male (based on 17 mature specimens)

 Body 4.27–7.02 (mean 5.83) mm long; maximum width 251–444 (356) mm. Esophagus 397–477 (429) mm long, representing between 5.76% and 9.31% (7.77%) of body length; corpus 275–304 (293) mm long; size of bulb 98–138 (116) × 143–180 (160) mm. Nerve-ring and excretory pore 159–203 (180) mm and 295–343 (310) mm from cephalic cap, respectively. Posterior end of body distinctly curved ventrally. Spicules ventrally bent, similar and subequal in length, distal end somewhat blunt (Figs. 1b, 3c, g), surface of spicules ornamented with randomly scattered punctations, extending through most of its length and disappearing near the tip (Fig. 1b, h); right spicule 388–550 (451) mm long, representing between 6.28% and 11.7% (8.12%) of body length; left spicule 363–525 (419) mm long, representing between 5.65% and 9.96% (7.53%) of body length. Gubernaculum absent. Caudal papillae 11 pairs: 4 pairs of precloacal (1st–3rd pairs ventro-lateral, 4th pair ventral) (Fig. 1b, c); 7 pairs of postcloacal papillae (5 pairs ventro-lateral, 2 pairs lateral) (Fig. 1b, d, e). Single medio-ventral, precloacal papillae present (Fig. 1b, e, f). Tail 125–205 (178) mm long, with short or long finger-like tip, representing between 2.44% and 4.28% (3.06%) of body length (Figs. 1b, e, 3c, h). Phasmids very small, between two postcloacal lateral papillae (Fig. 1e).

#### Female (based on 17 mature specimens)

 Body 6.00–9.60 (7.51) mm long; maximum width 304–629 (472) mm. Esophagus 403–623 (522) mm long, representing between 5.57% and 9.74% (7.36%) of body length; corpus 295–363 (326) mm long; size of bulb 100–148 (131) × 145–210 (177) mm. Nerve-ring and excretory pore 155–246 (196) mm and 275–367 (329) mm from cephalic cap, respectively. Vulva slit-like, post-equatorial, anterior vulval lip with remarkable flap (Fig. 2d, e, 3b, d), between 3.49 and 5.93 (4.44) mm from cephalic cap, representing between 53.2% and 76.0% (59.7%) of body length. Type II genital tract according to Adamson [9], consisting of a muscular, anteriorly directed, thick-walled vagina (Fig. 3b, d). Ovaries reflexed, didelphic amphidelphic. Eggs oval, thick-shelled, with smooth surface, unembryonated, 60–113 (90) × 58–75 (64) mm (*n* = 17) (Figs. 2f, 3b, d). Tail 163–248 (203) mm long, with polymorphic tip, representing between 2.09% and 3.87% (2.69%) of body length (Figs. 2c, 3d–g). Phasmids very small, at about posterior 1/3 of tail (Fig. 2c).

### Molecular characterization

#### Partial 18S region

Ten 18S sequences, of *R. sinense* n. sp., 877 bp in length, were obtained, with no nucleotide polymorphism detected. There is only one 18S sequence (KX844642.1) of *R. naylae* available in GenBank. Pairwise comparison of the 18S sequences of *R. naylae* obtained herein with that available in GenBank indicated 100% similarity. In the superfamily Rhigonematoidea, 18S sequences are also available in GenBank for *Rhigonema ingens* (JX069475.1), *Rhigonema thysanophora* (EF180067.1), *Xystrognathus phrissus* (JX101957.1), *Ichthyocephaloides sumbatus* (JX101958.1), *Obainia* sp. (KU561101.1) and *Trachyglossoides* sp. (MW030192.1). Pairwise comparison of the 18S sequences of *R. sinense* n. sp. with those available in GeneBank showed nucleotide divergence of 0.34% (*R. ingens*) to 6.74% (*Trachyglossoides* sp.).

#### Partial 28S region

Ten 28S sequences of *R. sinense* n. sp., 767 bp in length, were obtained, representing four different genotypes, which exhibited 0.13–0.26% nucleotide divergence. A limited number of 28S sequences of *R. naylae* are available in GenBank (KX844643, MT988354.1–MT988371.1). Pairwise comparison of the 28S sequences of *R. sinense* n. sp. with those available in GenBank showed 0.40–0.53% nucleotide divergence. In the superfamily Rhigonematoidea, 28S sequences are also available in GenBank for *R. ingens* (JX131616.1), *R. thysanophora* (MG195996.1), *X. phrissus* (JX155274.1), *I. sumbatus* (JX155273.1), *Obainia* sp. (KU561100.1) and *Trachyglossoides* sp. (MW030188.1). Pairwise comparison of the 28S sequences of *R. sinense* n. sp. with those available in Genbank showed nucleotide divergence of 3.31% (*R. ingens*) to 15.8% (*Trachyglossoides* sp.).

#### Partial ITS region

Nine ITS sequences of *R. sinense* n. sp., 1190–1191 bp in length, were obtained, representing eight different genotypes, which exhibited 0.084–0.76% nucleotide divergence. In the superfamily Rhigonematoidea, no species with ITS sequences are available in GenBank. Consequently, we sequenced the ITS region of *R. naylae* based on specimens collected from *P. tonominea* in Japan. Pairwise comparison of the ITS sequences of *R. sinense* n. sp. with that of *R. naylae* obtained in this study showed 1.57–2.69% nucleotide divergence.

#### Partial* cox*1 region

Ten *cox*1 sequences of *R. sinense* n. sp. were obtained, all 670 bp in length, representing six different genotypes, which exhibited 0.15–0.60% nucleotide divergence. In the superfamily Rhigonematoidea, *cox*1 sequences are available in GenBank only for *R. thysanophora* (NC_024020.1) and *Ru. karukerae* (MF509850.1). Pairwise comparison of the *cox*1 sequences of *R. sinense* n. sp. with those of *R. thysanophora* and *Ruizia karukerae* showed 19.8–22.9% and > 30% nucleotide divergence, respectively. In the present study, we also sequenced the *cox*1 region of *R. naylae* based on specimens collected from *P. tonominea* in Japan. Three *cox*1 sequences of *R. naylae* were obtained, all 676 bp in length, with no nucleotide polymorphism detected. Pairwise comparison of the *cox*1 sequences of *R. sinense* n. sp. with those of *R. naylae* displayed 14.3–14.9% nucleotide divergence.

#### Partial* cox*2 region

Ten *cox*2 sequences of *R. sinense* n. sp. were obtained, all 676 bp in length, representing three different genotypes, which exhibited 0.15–0.44% nucleotide divergence. In the superfamily Rhigonematoidea, *cox*2 sequences are available in GenBank only for *R. thysanophora* (NC_024020.1) and *Ru. karukerae* (MF509850.1). Pairwise comparison of the *cox*2 sequences of *R. sinense* n. sp. with those of *R. thysanophora* and *Ru. karukerae* displayed > 30% nucleotide divergence for both species. In the present study, we also sequenced the *cox*2 region of *R. naylae* based on specimens collected from *P. tonominea* in Japan; two *cox*2 sequences of *R. naylae* were obtained, both 530 bp in length, with no nucleotide polymorphism detected. Pairwise comparison of the *cox*2 sequences of *R. sinense* n. sp. with those of *R. naylae* obtained in this study displayed 13.0–13.3% nucleotide divergence.

#### Species delimitation

**Fig. 4:**
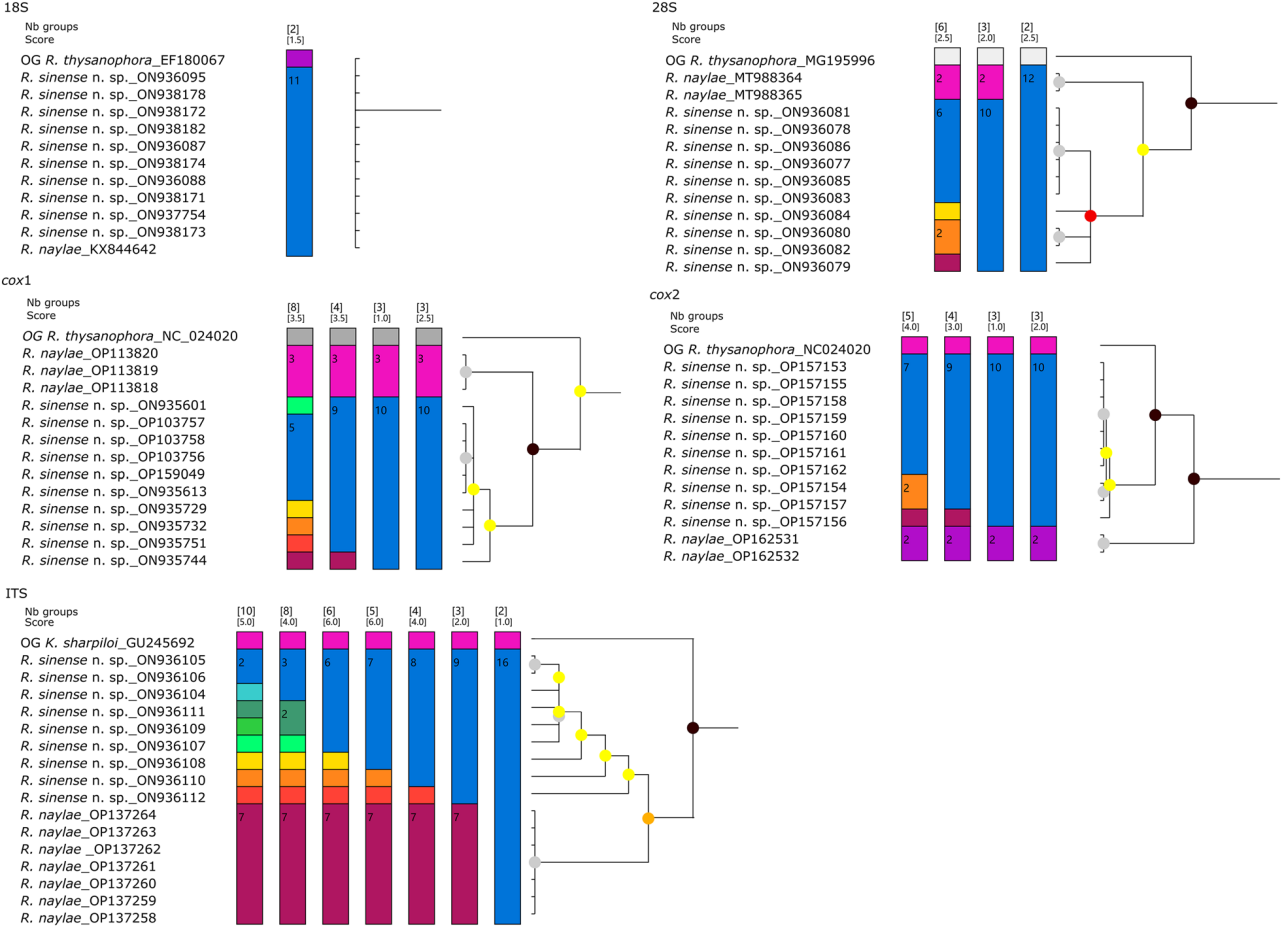
Assemble Species by Automatic Partitioning (ASAP) analyses of *Rhigonema sinense* n. sp. and *R. naylae* based on five different nuclear and mitochondrial genetic markers. Asterisk indicates the optimal result recommended by ASAP. cox1/2, Cytochrome* c* oxidase subunit ½; ITS, internal transcribed spacer; OG, out-group; 18S/28S, small/large ribosomal subunit

**Fig. 5:**
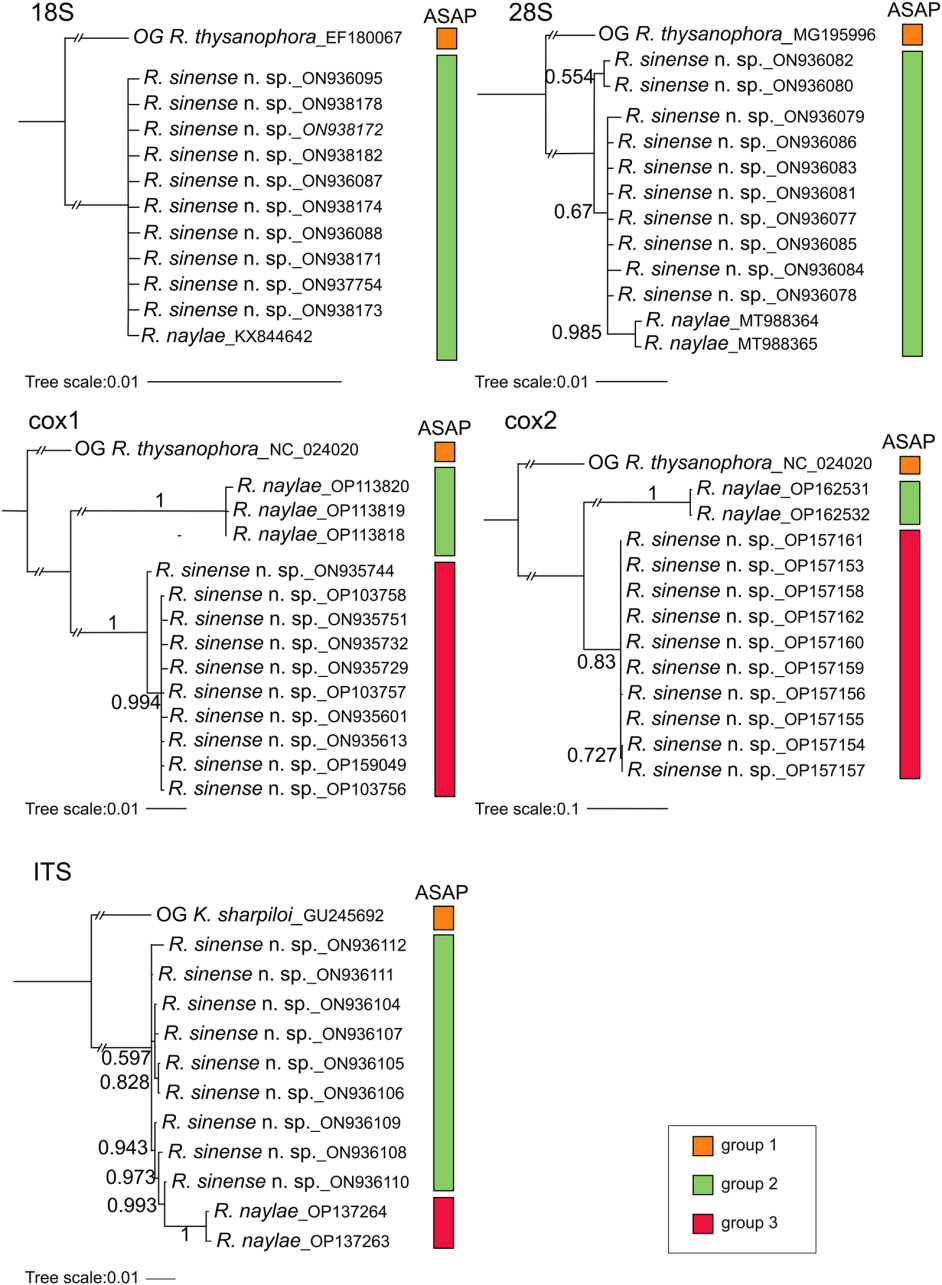
Bayesian inference and ASAP analyses of *Rhigonema sinense* n. sp. and *R. naylae* based on five different nuclear and mitochondrial genetic markers. Bayesian posterior probabilities values ≥ 0.70 are shown on nodes and ASAP results are shown on the right. Different colors represent different groups

All ASAP analyses based on the 28S, ITS, *cox*1 and *cox*2 sequences supported the species partition of *R. sinense* n. sp. and *R. naylae* (Figs. 4, 5). However, BI analyses based on the ITS and 28S sequences displayed *R. naylae* nested in samples of *R. sinense* n. sp. (Fig. 5). Our results of ASAP and BI analyses based on the *cox*1 and *cox*2 genes were concordant, which clearly showed that *R. sinense* n. sp. represents a separated species from *R. naylae* (Fig. 5). Moreover, ASAP analyses based on the 28S, ITS, *cox*1 and *cox*2 sequences showed no evidence that the different morphotypes of *R. sinense* n. sp. represent distinct genetic lineages (Fig. 4). The results of BI and ASAP analyses based on the 18S gene both showed that *R. sinense* n. sp. and *R. naylae*. formed a single group (Fig. 5).

#### Phylogenetic analyses

**Fig. 6:**
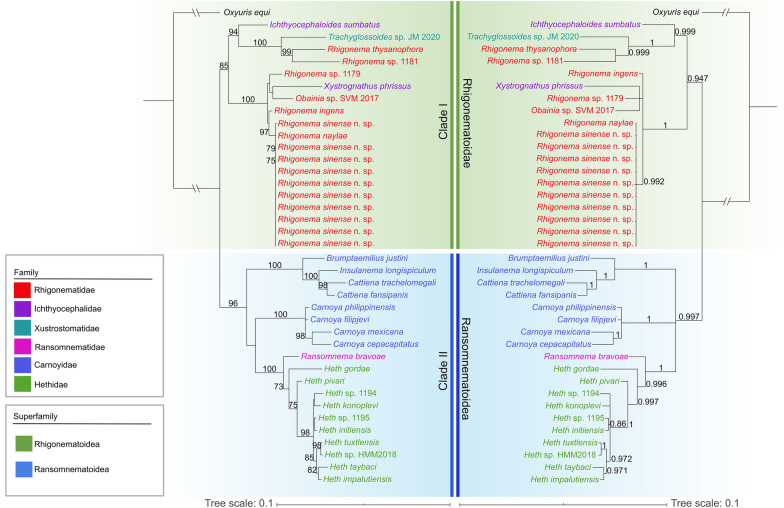
Phylogenetic relationships of representatives of the Rhigonematomorpha using maximum likelihood and Bayesian inference analyses based on the 18S + 28S sequences. *Oxyuris equi* (Oxyuridomorpha: Oxyuridae) was chosen as the out-group. Bootstrap values ≥ 70 and Bayesian posterior probabilities values ≥ 0.70 are shown on nodes in the phylogenetic trees

Phylogenetic trees constructed based on the 18S + 28S sequence data using ML and BI methods were nearly identical in topology, with both supporting the representatives of Rhigonematomorpha divided into two large clades (clade I and clade II) (Fig. 6). In the ML tree, clade I included species of the genera *Rhigonema*, *Ichthyocephaloides*, *Xystrognathus*, *Obainia* and *Trachyglossoides*, which represent the superfamily Rhigonematoidea. In clade I, the genera *Ichthyocephaloides* and *Xystrognathus* of the family Ichthyocephalidae did not cluster together (species of *Ichthyocephaloides* showed a sister relationship with *Trachyglossoides* sp. + *Rhigonema thysanophora* + *Rhigonema* sp. 1181, and *X. phrissus* clustered together with *Obainia* sp. SVM2017). The genus *Rhigonema* was not monophyletic as its representatives were present in some different and far lineages (Fig. 6).

Clade II was formed by species of the genera *Ransomnema*, *Carnoya*, *Brumptaemilius*, *Insulanema*, *Cattiena* and *Heth*, which represents the superfamily Ransomnematoidea. The representatives of the family Carnoyidae were divided into two separated branches with strong support in both the ML and BI trees (Fig. 6). Species of the genera *Brumptaemilius*, *Insulanema* and *Cattiena* clustered together, and the genus *Carnoya* was sister to representatives of the families Ransomnematidae and Hethidae with weak support (Fig. 6). However, in the BI tree, species of *Brumptaemilius*, *Insulanema* and *Cattiena* grouped together, forming three polyphyletic branches with species of *Carnoya* and the representatives of the families Ransomnematidae and Hethidae (Fig. 6). The family Ransomnematidae (*Ransomnema bravoae*) was sister to the Hethidae (*Heth* spp.), with strong support in both ML and BI trees.

## Discussion

The genus *Rhigonema* Cobb, 1898 is the largest group in the superfamily Rhigonematoidea, including approximately 90 nominal species that are mainly parasitic in millipedes in Africa, Asia, Australia and South and North America [4, 13, 17, 54–57]. Among the congeners, *R. sinense* n. sp. has its anterior 1/4 of body covered by spine, four pairs of precloacal papillae and seven pairs of postcloacal papillae, spicules subequal and not exceeding 0.60 mm in length, a relatively short tail tip in both sexes and the type 2 genital tract in females. Based on these characteristics, *R. sinense* n. sp. resembles the following* Rhigonema* species: *R. disparovis* Van Waerebeke, 1991, *R. fecundum* Hunt, 2002, *R. ingens* Hunt, 1998, *R. longicorpus* (Rao, 1973), *R. naylae* Morffe & Hasegawa, 2017, *R. neyrae* Singh, 1955, *R. ornatum* Majumdar, 1967, *R. oxydesmi* Hunt, 2002, *R. rigonanae* Hunt, 1999*, R. rostrellum* Hunt, 2002, *R. seychellarurn* Adamson, 1987, *R. spiridonovi* Hunt, 1999 and *R. trichopeplum* Hunt & Moore, 1995 [9, 11, 12, 17, 54, 56, 58–61].

*Rhigonema sinense* n. sp. differs from *R. fecundum*, *R. oxydesmi*, *R. rigonanae*, *R. rostrellum*, *R. spiridonovi* and *R. trichopeplum* by having markedly longer spicules (0.36–0.55 mm in the former species vs 0.22–0.34 mm in the latter six species). With three separated bilobed lips, *R. seychellarurn* can be easily distinguished from *R. sinense* n. sp. that has three lips fused together. The new species is also different from *R. disparovis*, *R. longicorpus*, *R. neyrae*, *R. naylae* and *R. ornatum* by having a remarkable anterior vulval flap in females (vs anterior vulvar flap absent in the latter five species). *Rhigonema sinense* n. sp. is most similar to *R. ingens* in morphometry and morphology; however, males of *R. ingens* are slightly longer (7.00–7.50 mm vs 4.27–7.02 mm in the new species). Moreover, we found the presence of 3.31% nucleotide divergence in the partial 28S gene between *R. sinense* n. sp. and *R. ingens*, which supported our present specimens representing a separated species from *R. ingens*.

Although some previous taxonomical studies provided 18S and/or 28S genetic data for diagnosis of species [5, 17, 18, 62–64], the molecular identification of Rhigonematomorpha nematodes remains in its beginning phase. BI and ASAP analyses based on the *cox*1 and *cox*2 sequences both supported species partition of *R. sinense* n. sp. and *R. naylae*. However, the results of BI inference and ASAP analyses of *R. sinense* n. sp. and *R. naylae* showed that the 18S gene, with its slow evolutionary rate, is unsuitable for species delimitation of Rhigonematomorpha nematodes.

The ITS sequences of *R. sinense* n. sp. and *R. naylae* were also provided in the present study. This is the first characterization of the ITS region for Rhigonematomorpha nematodes. Although the ASAP analyses based on both the ITS and 28S data supported the species partition of *R. sinense* n. sp. and *R. naylae*, BI showed that *R. naylae* nested in samples of *R. sinense* n. sp. The results of the BI inference and ASAP analyses performed in the present study provide more convincing evidence that *R. sinense* n. sp. represents a separated species from *R. naylae*. Moreover, none of the different morphotypes of *R. sinense* n. sp. formed a monophyletic/separated group in the BI or ASAP analyses. There is no evidence that the different morphotypes of *R. sinense* n. sp. represent distinct genetic lineages. We considered the striking morphological variability in the tail tip of different individuals of *R. sinense* n. sp. as intraspecific variation.

Current knowledge of the molecular phylogeny of Rhigonematomorpha remains very limited. Although some previous molecular phylogenetic studies made some attempts to solve the evolutionary relationships of Rhigonematomorpha and its related taxa (i.e. Ascaridomorpha, Spiruromorpha and Oxyuridomorpha), as well as the systematic status of some families or genera in the Rhigonematomorpha [3, 5, 6, 15, 16, 22–24, 65], the basic molecular phylogenetic framework for the Rhigonematomorpha is far from complete. The phylogenetic results of the present study are largely congruent with the traditional classifications of the Rhigonematomorpha [1, 2, 4], which support the division of this taxon into two superfamily Rhigonematoidea and Ransomnematoidea. Our results are also in agreement with those of a previous molecular phylogenetic study based on 18S + 28S sequence data [3], but conflict with some molecular phylogenies using single 18S or 28S sequence data [5–7, 19].

The phylogenetic analyses performed in the present stuy indicate that the family Ichthyocephalidae and the genus *Rhigonema* in Rhigonematoidea are not monophyletic, which is consistent with the findings of previous studies [3, 5–7]. It is surprising that *R. thysanophora* + *Rhigonema* sp. were closely related with the family Xustromatidae (*Trachyglossoides* sp.), since species of *Rhigonema*, for example *R. thysanophora*, have a very different morphology of cephalic end and esophagus when compared with members of the Xustromatidae [66, 67]. The evolutionary relationships of the three families Rhigonematidae, Ichthyocephalidae and Xustromatidae in the Rhigonematoidea remain unsolved.

In the superfamily Ransomnematoidea, a previous study showed that the Ransomnematidae has a sister relationship with the Hethidae with weak support [3]. However, our phylogenetic results support the Ransomnematidae as having a sister relationship with the Hethidae with strong support in both the ML and BI trees. According to Poinar [68], the Carnoyidae includes only *Carnoya* and *Rondonema*. Subsequently, the genera *Brumptaemilius*, *Clementeia*, *Raonema*, *Urucuia* and *Waerebekeia* were transferred into the Carnoyidae [4]. Recently, two newly erected genera. *Insulanema* and *Cattiena*, were placed into the Carnoyidae [5, 69]. However, the phylogenetic results showed that the Carnoyidae, with representatives of *Carnoya*, *Brumptaemilius*, *Insulanema* and *Cattiena*, is not a monophyletic group. We strongly support the resurrection of the family Brumptaemiliidae for *Brumptaemilius*, *Insulanema* and *Cattiena*. A more rigorous molecular phylogenetic study that includes broader representatives of the Rhigonematomorpha using more nuclear and mitochondrial sequence data is need to further ascertain the phylogenetic relationships of different families.

## Conclusions

A new species of Rhigonematomorpha, *R. sinense* n. sp., is described based on specimens collected from *S. bungii* in China. ASAP analyses using 28S, ITS, *cox*1 and *cox*2 data support the species partition of *R. sinense* n. sp. and *R. naylae*, and also indicate the striking variability in tail morphology of *R. sinense* n. sp. as intraspecific variation, in turn suggesting that the partial 28S, ITS, *cox*1 and *cox*2 regions are effective for molecular identification of Rhigonematomorpha nematodes. Moreover, the molecular phylogenetic results of our study support the traditional classification of the infraorder Rhigonematomorpha divided into two superfamilies, Rhigonematoidea and Ransomnematoidea, and also show that the families Carnoyidae, Ichthyocephalidae and the genus *Rhigonema* are non-monophyletic. The phylogeny reported here suggests that the Ransomnematidae is sister to the Hethidae, and that the family Brumptaemiliidae should be resurrected. However, the evolutionary relationships of three families within Rhigonematoidea, namely Rhigonematidae, Ichthyocephalidae and Xustromatidae, remain unresolved.

## Data Availability

The nuclear and mitochondrial DNA sequences of *Rhigonema sinense* n. sp. and *R. naylae* obtained in the present study were deposited in GenBank database (sequences of *R. sinense* under the accession numbers: ON936087, ON936088, ON936095, ON937754, ON938171–ON938174, ON938178, ON938182 (18S), ON936077–ON936086 (28S), ON936104–ON936112 (ITS), OP159049, OP103756–OP103758, ON935601, ON935613, ON935729, ON935732, ON935744, ON935751 (*cox*1) and OP157153–OP157162 (*cox*2); sequences of *R. naylae* under the accession numbers: OP137258–137264 (ITS), OP113818–113820 (*cox*1), OP162531-162532) (*cox*). Type specimens of *R. sinense* n. sp. were deposited in the College of Life Sciences, Hebei Normal University, Hebei Province, China (under the accession numbers HBNU-N-2022Ar008Z-L, HBNU-N-2022Ar009Z-L and HBNU-N-2022Ar010Z-L).

## Abbreviations

ASAP: Assemble Species by Automatic Partitioning
BI: Bayesian inference
BIC: Bayesian information criterion
*cox*1: Cytochrome* c* oxidase subunit 1
*cox*2: Cytochrome* c* oxidase subunit 2
ITS: Internal transcribed spacer
ML: Maximum likelihood
18S: Small ribosomal subunit
28S: Large ribosomal subunit
SEM: Scanning electron microscopy
